# Evaluating on-line health information for patients with polymyalgia rheumatica: a descriptive study

**DOI:** 10.1186/s12891-017-1416-5

**Published:** 2017-01-26

**Authors:** Arani Vivekanantham, Joanne Protheroe, Sara Muller, Samantha Hider

**Affiliations:** 1 Arthritis Research UK Primary Care Centre, Research Institute for Primary Care and Health Sciences, Keele University, Staffordshire, ST5 5BG UK; 2 Haywood Rheumatology Centre, Haywood Hospital, Staffordshire, UK

**Keywords:** Polymyalgia rheumatica, Health literacy, Websites, Readability

## Abstract

**Background:**

The Internet is increasingly used to access health information, although the quality of information varies. The aim of this study was to evaluate the readability, and quality of websites about polymyalgia rheumatica (PMR).

**Methods:**

Three UK search engines (Google, Yahoo and Bing) were searched for the term ‘polymyalgia rheumatica’. After deleting duplicates, the first 50 eligible websites from each were evaluated. Readability was assessed using the Flesch Reading Ease and ‘Simple Measure of Gobbledygook (SMOG) Readability’ indicators. Credibility was assessed using a previously published Credibility Indicator.

**Results:**

Of the 52 unique websites identified, the mean (standard deviation) Flesch Reading Ease and SMOG Readability scores were 48 (15) and 10 (2), respectively. The mean (SD) Credibility Indicator was 2 (1). Fifty (96%) of websites were accurate. Website design and content was good, with an average of 68 and 64% respectively, of the assessed criteria being met.

**Conclusions:**

Most websites about PMR require a higher readability age than is recommended. Thus whilst websites are often well designed and accurate this study suggests that their content could be refined and simplified to maximise patient benefit.

## Background

‘Health literacy’ refers to the ability to perform basic reading and numerical tasks required to function effectively in the healthcare environment. This includes the ability to read, understand and interpret information (print literacy), perform quantitative tasks such as following treatment regimens (numeracy) and to speak and listen effectively (oral literacy) [1]. Low levels of health literacy are associated with poorer disease control, increased health care costs [2] and increased mortality [3].

Therefore, provision of good quality yet accessible health information is increasingly important, especially in the management of long term conditions. A longitudinal study found that one third of respondents aged 63 to 66 years had searched for on-line information about their health [4]. The unrestricted nature of the internet means that evaluating sites for quality and content is increasingly important.

However, health information is only useful if patients can read, understand and apply it to their own circumstances. Studies show that low literacy levels are common [5], leading to guidelines for patient information to be written at sixth grade level (age 11 to 12 years) [6, 7] to maximise accessibility. Quantitative readability measures such as the Flesch Reading Ease [8] and Simple Measure of Gobbledygook (SMOG) Readability [9] tools have been developed to evaluate the appropriateness of written health information [10]. Furthermore in addition to being able to read and understand information, information should be both accurate and accessible. Studies suggest that appropriate presentation of information on the internet (eg by using bulleted lists rather than large passages of text) can enhance its usability and as such specific usability and credibility (indicators have been developed [11].

There is growing interest in the importance of patient health literacy in long term conditions. Studies evaluating patient education materials for rheumatological conditions including osteoarthritis, rheumatoid arthritis, systemic lupus erythematosus and vasculitis found many to be written at readability levels above the recommended sixth-grade reading level [10].

Given the chronic nature of polymyalgia rheumatica (PMR), patients and their carers may be more likely to seek additional health information via the internet. To date studies have not evaluated internet website resources for patients, especially whether they are designed at appropriate readability levels and therefore likely to be understood by users. Therefore, the aim of this study was to evaluate the readability, credibility and usability, design and content of websites for PMR.

## Methods

### Identification of websites

The three most commonly used UK search engines (Google, Yahoo and Bing) were searched for the term ‘polymyalgia rheumatica’ on 31 July 2013. The search page results from each of the search engines were saved in a PDF format, with a hyperlink for each search page and website to ensure that the same pages found in this original search could be accessed again. Starting with the highest ranking website, the first 50 eligible websites from each search engine were evaluated. Websites were excluded if they were videos, chat forums or product advertisements, or if it clearly stated that it was intended solely as a professional resource. Websites were evaluated only once if they were identified by more than one search engine. Excluded websites were replaced by the next eligible website found by the search engine, leaving 50 sites from each search engine. No human data is used and therefore ethical approval is not required.

### Readability

Readability was measured using the Flesch Reading Ease [8] and SMOG Readability [9] tools. The Flesch Reading Ease tool measures readability using a formula that assesses word and sentence length. It rates text on a 100-point scale; the greater the score, the easier it is to understand. A Flesch readability score of 60 or above is considered to be easy to follow. This tool has a high correlation to other readability scales, excellent reproducibility and has been used in numerous studies. The ‘SMOG Readability’ tool also measures readability using a formula, from 30 sentences (10 from the start, 10 from the middle and 10 from the end of the text of interest), counting the number of words containing three or more syllables. The SMOG score is the square root of the total word count plus 3. A score between 3–8, 9–12 and 13 or more indicates that completion of primary, secondary and tertiary education respectively is needed in order to comprehend the information. This tool is simple to use, repeatable and accurate in determining the reading level.

For the readability assessment of the websites, the first 600 words of the website content was copied and pasted into a free on-line text readability calculator to calculate the ‘Flesch Reading Ease’ and ‘SMOG Readability’ scores. Titles, subtitles, references, web links and advertising text were excluded from the readability analysis, with only body text and bullet point text included. Although the first 600 words of the website were assessed for readability the rest of the site was examined for credibility, usability and content as described below.

### Website credibility and quality

Website credibility and usability was assessed using the 22 variables described by [11], which was designed to comprise variables easily identifiable and interpreted by people irrespective of their level of education. From this we calculated the 8-item Credibility Indicator described (incorporating authorship, affiliation, editorial team, date of creation, date of update, backing, accreditation and financing).

### Website design

To assess website design two previously published criteria which include features to make the site easier to read (e.g. font sizes, use of bulleted text) and use of images were combined [12, 13].

### Content accuracy

Information contained on each site was summarised into clinical domains PMR (e.g. symptoms and signs, management, prognosis) and accuracy assessed by clinicians (including a consultant rheumatologist (SH) and general practitioner (JP)).

### Statistical analyses

The mean and standard deviation (SD) of the readability scores (‘Flesch Reading Ease’ and ‘SMOG Readability’) was calculated across all websites assessed. For other domains, the proportion of websites scoring positively on each item was calculated and the 8 item Credibility Index calculated. Results are presented as mean (SD) unless otherwise stated.

## Results

**Fig. 1:**
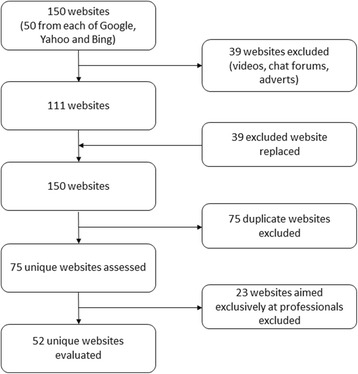
Website selection process from the three search engines

Figure 1 shows the website selection process from the three search engines. After removing duplicate sites and those aimed purely at healthcare professionals, 52 sites remained for evaluation.

### Readability

The mean (SD) ‘Flesch Reading Ease’ and ‘SMOG Readability’ scores of the websites were 48 (15) and 10 (2), respectively.

### Credibility and usability

Table 1 details the credibility and quality results. The mean (SD) Credibility Indicator was 2 (1). 36 (69%) websites included contact details and 42 (81%) had a built-in search facility. Information regarding financial support, date of creation and named authors were only included in 1 (2%), 8 (15%) and 11 (21%) of websites, respectively. Moreover, appropriate bodies accredited only 11 (21%) of the websites and only 8 (15%) included a ‘help’ option. The ability to change the font size was available for 14 (27%) of websites.

**Table 1:** Website readability and content

Variable	Measure	*n* (%) meeting criteria^a^
Readability	The Flesch Reading Ease Readability	Mean (SD): 48 (15)
	SMOG Readability	Mean (SD): 10 (2)
Content	Epidemiology	23 (44)
	Aetiology	33 (63)
	Symptoms	49 (94)
	Signs	46 (88)
	Investigations	39 (75)
	Criteria for diagnosis	28 (54)
	Management	50 (96)
	Prognosis	16 (31)
	Differential diagnosis	12 (23)
	Is the content of the website accurate, to your knowledge?	45 (87)

^a^Unless otherwise stated

### Design

Design of the websites was generally good. All used consistent designs, font sizes and styles with 51 (98%) using a font size of at least 12-points and 44 (85%) of websites ‘chunking’ information into meaningful sections with clear headings. Moreover, 47 (90%) used short sentences and an active voice, with 45 (87%) avoiding the use of jargon or technical language. However, only 5 (7%) included video or audio illustration and only 8 (15%) supplemented the text with illustrations.

### Content

Evaluation of all 52 websites found variation in the type of content (Table 2). Whilst 49 (94%) provided information regarding symptoms and all included aspects of management, fewer provided information on prognosis (*n* = 16, 31%) although most sites were accurate (*n* = 50, 96%), with some important inaccuracies including using herbal supplements as treatment for PMR, and others suggesting statins cause PMR. Furthermore although many websites highlighted the link between PMR and GCA only 44% had appropriate advice regarding seeking urgent medical attention if visual symptoms developed, and only 25% contained appropriate advice for what to do about steroids if unwell, suggesting that some key patient messages are not universally highlighted.

**Table 2:** Website credibility, quality and design

Variable	Measure	*n* (%) meeting criteria^a^
Credibility and Quality [11]	Authorship^b^	11 (21)
	Affiliation^b^	46 (88)
	Editorial team^b^	10 (19)
	Date of creation^b^	8 (15)
	Date of update^b^	19 (37)
	Backing^b^	13 (25)
	Accreditation^b^	11 (21)
	Financing^b^	1 (2)
	Credibility Indicator	Mean (SD): 2 (1)
	Coherence of the title	51 (98)
	Contact	36 (69)
	Validity of the links (first 3)	48 (92)
	Coherence of the links	50 (96)
	Help	8 (15)
	Font size	14 (27)
	Information management	28 (54)
	Declaration of conflict of interests	0 (0)
	Objectivity	0 (0)
	Site traffic statistics	0 (0)
	Website search engine	42 (81)
	Accessibility	52 (100)
	Interoperability	52 (100)
	Editorial policy	0 (0)
Design [12, 13]	Use of consistent designs, font sizes, and styles	52 (100)
	Video/audio illustration	5 (7)
	Use a sans serif font for text	51 (98)
	Use of short sentences and an active voice	47 (90)
	Avoidance of jargon/technical language	45 (87)
	Use bulleted lists rather than large blocks of text	20 (38)
	Q&A section	4 (8)
	Use at least a 12-point font	51 (98)
	‘Chunk’ information into meaningful sections with clear headings	44 (85)
	Leave right margins jagged (i.e. do not justify)	52 (100)
	Avoid percentages (‘one out of ten’ rather than ‘10%’)	18 (35)
	Use numerals rather than spelling out numbers	46 (88)
	Supplement with illustrations	8 (15)
	Use of upper and lower case (avoids using all capital letters, italics, and fancy script)	50 (96)

^a^Unless otherwise stated

^b^Variables included in the Credibility Indicator [11]

## Discussion

With the increasing number of people using the internet to access health-related information, it is essential that the information on websites is readable, accurate, credible and user-friendly. Whilst the accuracy and design of websites providing information on PMR is generally good, although there are some key omissions, this study highlights that the readability of these sites is poor, with the majority of the websites having a reading age of at least 16 years, significantly higher than the United States Department of Health and Human Services (USDHHS) recommended reading age of 10–12 years for patient information [6], suggesting their effectiveness could be improved to ensure they are widely accessible. These findings are in line with those reported previously, suggesting that it is common for patient health information to require higher than the recommended reading ages [10]. Given that health literacy levels are known to be lower in older people than in the general population [14], this may be a particular issue for patients with PMR, suggesting that significant revisions may be needed to ensure that information is accessible.

These findings support the proposal by Fitzsimmons et al. encouraging website editors to consider introducing a minimum readability policy based on the USDHHS guidelines [6], using a validated readability measure to improve the comprehension of patient information such as the ‘SMOG Readability’ measure, which is easy to use and for which on-line calculators are available [15].

In addition to the poor readability of the PMR websites, this study found that the credibility and usability of most of the PMR patient orientated websites could be improved. Many did not state the date of creation, accreditation or detail authors making it difficult to assess how up to date these sites are or who wrote them. Moreover, the majority of the websites did not have a ‘help’ option or have the ability to change the size of the text, suggesting that these websites have not taken into account those patients who are visually impaired, which may be a particular problem for older adults.

A key strength of this study is that since it evaluated the first 50 websites from three of the most commonly used search engines in the UK, it is likely to have assessed those websites that patients are likely to read. Moreover, this study not only evaluated the readability, credibility and usability of the websites, but also their design and content. This is in contrast to most other studies that have looked at on-line health information with regards to a particular condition, that have tended to focus on evaluating one specific aspect of website quality [11, 15].

## Conclusions

In summary, although there is a wide range of PMR websites, this study suggests that many require a higher reading age than recommended. This suggests that the readability, credibility and usability of PMR websites should be reconsidered to maximise their likely patient benefit.

## Abbreviations

PMR: Polymyalgia rheumatica
SD: Standard deviation
SMOG: Simple Measure of Gobbledygook
USDHHS: United States Department of Health and Human Services
